# Treatment of Injuries of Joints

**Published:** 1918-12

**Authors:** 


***The Treatment of Injuries of the Joints in War-Surgery.*** By MM. Rouvillois and Guillaume-Louis. *Bulletin de la Société de Chirurgie de Paris*, May 14, 1918.

The authors had the opportunity of studying a good many cases of fractures of the joints. They consider three different types of these :

1) Metaphyseal primitive fractures followed by epiphyseal injury.

2) Epiphyseal fractures.

3) Epiphyseal primitive fractures, followed bymetaphyseal injury.

1) The initial lesion is in the metaphysis or even in the diaphysis, and extends to the joint by one or several fissures. Fractures of this kind occur frequently in the elbow.

2) These are real fractures of the joint; frequent in the inferior limbs. The whole injury is limited in the joint. They may be :

*a*) incomplete, only one of the epiphysis and one of the condyles being torn away, or

*b*) complete, both epiphysis being injured.

3) The projectile fractured first the joint, but the injury extended to the metaphysis or even to the diaphysis. These are the most severe cases.

For the treatment of the wound the authors distinguish :

1) *The Fractures by Bullets.* The wounds in these cases are generally aseptic and their treatment is simple. Surgical interventions shall take place if a projectile or some splinters have to be removed. Generally there is no infection, and after a complete cleansing and the realization of the hemostasis, the primitive suture can be performed.

2) *Fractures by Shell-fragments.* All general processes, such as : removal of projectiles, disinfection of the wound, complete hemostasis, must, of course, take place immediately. After these, the wound gets surgical treatment. In the beginning of the war, the primitive resection seemed to be the only way of preventing arthritis, and was practised constantly. Nowadays, arthrotomy has regained much favor. When practised with an appropriate incision it has good results, and permits the complete examination and disinfection of all synovial " culs-de-sacs ". Resection is only applied when it seems impossible to keep the loose fragments of bones.

In all cases the authors consider drainage of the wound not only useless but even very harmful. The synovial membrane must be sutured as soon as possible to prevent infection.

The wound will generally heal aseptically, its sterility being proved by bacteriological control; threads may be removed after 8 or 10 days. The joint must be carefully mobilized to avoid ankylosis.

— In cases of injuries of the shoulder, it sometimes proves difficult to realize a complete hemostasis. A drainage will then be necessary, but it must not last over 48 hours. —

3) *Complete Smashing of the Joint.* In those cases, the vessels and nerves are generally injured together with the bones and muscles. Amputation is generally unavoidable; it must be performed as soon as possible to prevent gangrene.

